# Supplementary material for: Heat adaptation of phage T7 under an extended genetic code
Source: Virus Evol. 2021 Dec 1;7(2):veab100. doi: 10.1093/ve/veab100 (PMC8923235; doi:10.1093/ve/veab100)
Supplement: veab100_Supp [file veab100_supp.zip › Sequencing_Analysis.zip!/Sequencing_Analysis/A2/evidence/JC_SIDE_1_51.html]

BRESEQ :: Evidence


| New junction evidence | | | | | | | | | | | |
| --- | --- | --- | --- | --- | --- | --- | --- | --- | --- | --- | --- |
|  | | seq id | position | reads (cov) | reads (cov) | score | skew | freq | annotation | gene | product |
| \* | ? | NC\_001604.1 | = 13455 | 2 (0.000) | 269 (0.600) | 158/586 | 0.3 | 94.6% | intergenic (–/–) | *–/–* | –/– |
|  | ? | NC\_001604.1 | 14331 = | 29 (0.070) |  |  |  |  | intergenic (–/–) | *–/–* | –/– |

Only 100 of 282 total aligned reads displayed.  

`GCTACATGGAATACAACAAGGAAACCGGATGGCTTGAACCATCAAGTTACTCAGGGGAAGAAGAGTCACACTCAGAGTCAACAGACTGGTCCAACGACACTGACTTCTGACAGGATTCTTGATGACTTTCCAGACGACTACGAGAAGTTTCGCTGGAGAGTCCCATTCTAATACGACTCACTAAAGGAGACACACCATGTTCAAACTG‑ATTAAGAAGTTAGGCCAACTGCTGGTTCGTATGTACAACGTGGAAGCCAAGCGACTGAACGATGAGGCTCGTAAAGAGGCCACACAGTCACGCGCTCTGGCGATTCGCTCCAACGAACTGGCTGACAGTGCATCCACTAAAGTTACCGAGGCTGCCCGTGTGGCAAACCAAGCTCAACAGCTTTCCAAATTCTTTGAGTAATCAAACAGGAGAAACCATTATGTCTAACGTAGCTGAAACTATCCGTCTATCCGATACAGCTGACCAGTGGAACCGTCGAGTCCACATCAACGTTCGCAACGGTAAGGCGACTATGGTTTACCGCTGGAAGGACTCTAAGTCCTCTAAGAATCACACTCAGCGTATGACGTTGACAGATGAGCAAGC  >  NC_001604.1/13156‑13750`  

                                                                                                                                                                                                                                                                                                            `|`                                                                                                                                                                                                                                                                                                         
`atgtgtataagagacagaaggaaaccggattgcttgAACCATCAAGTTACTCAGGGGAAGAAGAGTCACACTCAGAGTCAACAGACTGGTCCAACGACACTGACTTCTGACAGGATTCTTGATGACTTTCCAGACGACTACGAGAAGTTTCGCTGGAGAGTCCCATTCTAATACGACTCACTAAAGGAGACACACCATGTTCAAACTG‑ATTAAGAAGTTAGGCCAACTGCTGGTTCGTATGTACAACGTGGAAGCCAAGCGACTGAACGATGAGGCTCGTAAAGAGGCCACACAGTCACGa                                                                                                                                                                                                                                                                                                        <  1:76134‑M1/284‑2 (MQ=255)`  
   `aCATGGAATACAACAAGGAAACCGGATGGCTTGAACCATCAAGTTACTCAGGGGAAGAAGAGTCACACTCAGAGTCAACAGACTGGTCCAACGACACTGACTTCTGACAGGATTCTTGATGACTTTCCAGACGACTACGAGAAGTTTCGCTGGAGAGTCCCATTCTAATACGACTCACTAAAGGAGACACACCATGTTCAAACTG‑ATTAAGAAGTTAGGCCAACTGCTGGTTAGTATGTACAACGTGGAAGCCAAGCGACTGAACGATGAGGCTCGTAAAGAGGCCACACAGTCACGataa                                                                                                                                                                                                                                                                                                     <  1:662244‑M1/301‑5 (MQ=255)`  
                `caaGGAAACCGGATGGCTTGAACCATCAAGTTACTCAGGGGAAGAAGAGTCACACTCAGAGTCAACAGACTGGTCCAACGACACTGACTTCTGACAGGATTCTTGATGACTTTCCAGACGACTACGAGAAGTTTCGCTGGAGAGTCCCATTCTAATACGACTCACTAAAGGAGACACACCATGTTCAAACTG‑ATTAAGAAGTTAGGCCAACTGCTGGTTCGTATGTACAACGTGGAAGCCAAGCGACTGAACGATGAGGCTCGTAAAGAGGCCACACAGTCACGataatcaataggagctg                                                                                                                                                                                                                                                                                        >  1:670638‑M1/1‑284 (MQ=255)`  
                  `aGGAAACCGGATGGCTTGAACCATCAAGTTACTCAGGGGAAGAAGAGTCACACTCAGAGTCAACAGACTGGTCCAACGACACTGACTTCTGACAGGATTCTTGATGACTTTCCAGACGACTACGAGAAGTTTCGCTGGAGAGTCCCATTCTAATACGACTCACTAAAGGAGACACACCATGTTCAAACTG‑ATTAAGAAGTTAGGCCAACTGCTGGTTCGTATGTACAACGTGGAAGCCAAGCGACTGAACGATGAGGCTCATAAAGAGGCCACACAGTCACGataatcaataggagaaatc                                                                                                                                                                                                                                                                                      <  1:93207‑M1/301‑20 (MQ=255)`  
                  `aGGAAACCGGATGGCTTGAACCATCAAGTTACTCAGGGGAAGAAGAGTAACACTCAGAGTCAACAGACTGGTCCAACGACACTGACTTCTGACAGGATTCTTGATGACTTTCCAGACGACTACGAGAAGTTTCGCTGGAGAGTCCCATTCTAATACGACTCACTAAAGGAGACACACCATGTTCAAACTG‑ATTAAGAAGTTAGGCCAACTGCTGGTTCGTATGTACAACGTGGAAGCCAAGCGACTGAACGATGAGGCTCGTAAAGAGGCCACACAGTCACGataatcaataggagaaatc                                                                                                                                                                                                                                                                                      <  1:479240‑M1/301‑20 (MQ=255)`  
                       `aCCGGATGGCTTGAACCATCAAGTTACTCAGGGGAAGAAGAGTCACACTCAGAGTCAACAGACTGGTCCAACGACACTGACTTCTGACAGGATTCTTGATGACTTTCCAGACGACTACGAGAAGTTTCGCTGGAGAGTCCCATTCTAATACGACTAACTAAAGGAGACACACCATGTTCAAACTG‑ATTAAGAAGTTAGGCCAACTGCTGGTTCGTATGTACAACGTGGAAGCCAAGCGACTGAACGATGAGGCTCGTAAAGAGGCCACACAGTCACGataatcaataggagaaatcaatat                                                                                                                                                                                                                                                                                 <  1:579385‑M1/301‑25 (MQ=255)`  
                         `cGGATGGCTTGAACCATCAAGTTACTCAGGGGAAGAAGAGTCACACTCAGAGTCAACAGACTGGTCCAACGACACTGACTTCTGACAGGATTCTTGATGACTTTCCAGACGACTACGAGAAGTTTCGCTGGAGAGTCCCATTCTAATACGACTCACTAAAGGAGACACACCATGTTCAAACTG‑ATTAAGAAGTTAGGCCAACTGCTGGTTCGTATGTACAACGTGGAAGCCAAGCGACTGAACGATGAGGCTCGTAAAGAGGCCACACAGTCACGatactgtctcttatacacatctccga                                                                                                                                                                                                                                                                               >  1:620244‑M1/1‑275 (MQ=255)`  
                              `ggCTTGAACCATCAAGTTACTCAGGGGAAGAAGAGTCACACTCAGAGTCAACAGACTGGTCCAACGACACTGACTTCTGACAGGATTCTTGATGACTTTCAAGACGACTACGAGAAGTTTCGCTGGAGAGTCCCATTCTAATACGACTAAATAAAGGAGACACACCATGTTAAAACTG‑ATTAAGAAGTTAGGCCAACTGCTGGTTCGTATGTACAACGTGGAAGCCAAGCGACTGAACGATGAGGCTCGTAAAGAGGCCACACAGTCACGataatcaatcggagaaatcaatatgatcgtt                                                                                                                                                                                                                                                                          >  1:825827‑M1/1‑270 (MQ=255)`  
                              `ggCTTGAACCATCAAGTTACTCAGGGGAAGAAGAGTCACACTAAGAGTCAACAGACTGGTCCAACGACACTGACTTCTGACAGGATTCTTGATGACTTTCCAGACGACTACGAGAAGTTTCGCTGGAGAGTCCCATTCTAATACGACTCACTAAAGGAGACACACCATGTTCAAACTG‑ATTAAGAAGTTAGGCCAACTGCTGGTTCGTATGTACAACGTGGAAGCCAAGCGACTGAACGATGAGGCTCGTAAAGAGGCCACACAGTCACGataatcaataggagaaatcaatatgatcgtt                                                                                                                                                                                                                                                                          >  1:466872‑M1/1‑270 (MQ=255)`  
                               `gCTTGAACCATCAAGTTACTCAGGGGAAGAAGAGTCACACTCAGAGTCAACAGACTGGTCCAACGACACTGACTTCTGACAGGATTCTTGATGACTTTCCAGACGACTACGAGAAGTTTCGCTGGAGAGTCCCATTCTAATACGACTCACTAAAGGAGACACACCATGTTCAAACTG‑ATTAAGAAGTTAGGCCAACTGCTGGTTCGTATGTACAACGTGGAAGCCAAGCGACTGAACGATGAGGCTCGTAAAGAGGCCATACAGTCACGataatcaatctgtctcttatacacatctccga                                                                                                                                                                                                                                                                         >  1:690224‑M1/1‑269 (MQ=255)`  
                                   `ctcggagagtggtataagagacaggaagaGTCACACTCAGAGTCAACAGACTGGTCCAACGACACTGACTTCTGACAGGATTCTTGATGACTTTCCAGACGACTAAGAGAAGTTTCGCTGGAGAGTTACATTCTAAGACGACTCACTAAAGGAGACATAATATGTTCAAACTG‑ATTAAGAAGTTAGGCCAACTGCTGGTTAGTATGTACAACGTGGAAGCCACGCGACTGCACGATGAGGCTCGTAAAGAGGCCACACAGTCACGCGCTCTGGCGATTCGCTCCAACGAACTGGCTGACAg                                                                                                                                                                                                                                                                     <  1:103485/277‑1 (MQ=255)`  
                                            `aGTTACTCAGGGGAAGAAGAGTCACACTCAGAGTCAACAGACTGGTCCAACGACACTGACTTCTGACAGGATTCTTGATGACTTTCCAGACGACTACGAGAAGTTTCGCTGGAGAGTCCCATTCTAATACGACTCACTAAAGGAGACACACCATGTTCAAACTG‑ATTAAGAAGTTAGGCCAACTGCTGGTTCGTATGTACAACGTGGAAGCCAAGCGACTTAACTATGAGGCTCGTAAAGAGGCCACACAGTCACGataatcaataggagaaatcaatatgatcgtttctgacatcgaagc                                                                                                                                                                                                                                                            >  1:388178‑M1/1‑256 (MQ=255)`  
                                             `gTTACTCAGGGGAAGAAGAGTCACACTCAGAGTCAACAGACTGGTCCAACGACACTGACTTCTGACAGGATTCTTGATGACTTTCCAGACGACTACGAGAAGTTTCGCTGGAGAGTCCCATTCTAATACGACTCACTAAAGGAGACACACCATGTTCAAACTG‑ATTAAGAAGTTAGGCCAACTGCTGGTTCGTATGTACAACGTGGAAGCCAAGCGACTGAACGATGAGGCTCGTAAAGAGGCCACACAGTCACGCGCTCTGGCGATTCGCTCCAACGactgtctcttatacacatctccg                                                                                                                                                                                                                                                           >  1:188240/1‑279 (MQ=255)`  
                                                `aCTCAGGGGAAGAAGAGTCACACTCAGAGTCAACAGACTGGTCCAACGACACTGACTTCTGACAGGATTCTTGATTACTTTCCAGACGACTACGAGAAGTTTCGCTGGAGAGTCCCATTCTAATACGACTCACTAAAGGAGACACACCATGTTCAAACTG‑ATTAAGAAGTTAGGCCAACTGCTGGTTCGTATGTACAACGTGGAAGCCAAGCGACTGAACGATGAGGCTCGTAAAGAGGCCACACAGTCACGataatcaataggagaaatcaatatgatcgtttctgacatcgaagcctgt                                                                                                                                                                                                                                                        >  1:495713‑M1/1‑252 (MQ=255)`  
                                                   `cAGTGGAAGAAGAGTCACACTCAGAGTCAACAGACTGGTCCAACGACACTGACTTCTTACAGGATTCTAGATGACGTTCCAGACGACTACGAGAAGTTTCGCTGGAGAGTCCCATTCTAATACAACTCACTAAAGGAGACACACCATGTTCAAACTG‑ATTAAGAAGTTAGGCCAACTGCTGGTTCGTATGTACAACGTGGAAGCCAAGCGACTGAACGATGAGGCTCGTAAAGAGGCCACACAGTCACGataatcaataggagaaatcaatatgatcgtttctgacatcgaagctaacgcc                                                                                                                                                                                                                                                     <  1:230285‑M1/301‑53 (MQ=255)`  
                                                   `cAGGGGAAGAAGAGTCACACTCAGAGTCAACAGACTGGTCCAACGACACTGACTTCTGACAGGATTCTTGATGACTTTCCAGAAGACTACGAGAAGTTTCGCTGGAGAGTCCCATTCTAATACGACTCACTAAAGGAGACACACCATGTTCAAACTG‑ATTAAGAAGTTAGGCCAACTGCTGGTTAGTATGTACAACGTGGAAGCCAAGCGACTGAACGATGAGGCTCGTAAAGAGGCCACACAGTCACGataatcaataggagaaatcaatatgatcgtttctgacatcgaagctaacgcc                                                                                                                                                                                                                                                     <  1:736649‑M1/301‑53 (MQ=255)`  
                                                     `ggggAAGACGAGTCACACTCAGAGTCAACAGACTGGTCCAACGACACTGACTTCTGACAGGATTCTTGATGACTTTCCAGACGACTACGAGAAGTTTCGCTGGAGAGTCCCATTCTAATACGACTCACTAAAGGAGACACACCATGTTCAAACTG‑ATTAAGAAGTTAGGCCAACTGCTGGTTCGTATGTACAACGTGGAAGTCAAGCGACTGAACGATGAGGCCCGTAAAGAGGCCACACAGTCACGataatcaataggagaaatcaatatgatcgtttccgacatccaagctaccgactt                                                                                                                                                                                                                                                   >  1:77936‑M1/1‑247 (MQ=255)`  
                                                     `ggggAAGAAGAGTCACACTCAGAGTCAACAGACTGGTCCAACGACACTGACTTCTGACAGGATTCTTGATGACTTTCCAGACGACTACGAGAAGTTTCGCTGGCGAGTCCCATTCTAATACGACTCACTAAAGGAGACACACCATGTTCAAACTG‑ATTAAGAAGTTAGGCCAACTGCTGGTTCGTATGTACAACGTGGAAGACAAGCGACTGAACGATGAGGCTCGTAAAGAGGCCACACAGTAACGataatcaatcggagaactcaatatgatcgtttctgccatcgaagctaacgccct                                                                                                                                                                                                                                                   >  1:84562‑M1/1‑247 (MQ=255)`  
                                                     `ggggAAGAAGAGTCACACTCAGAGTCAACAGACTGGTCCAACGACACTGACTTCTGACAGGATTCTTGATGACTTTCCAGACGACTACGAGAAGTTTCGCTGGAGAGTCCCATTCTAATACGACTCACTAAAGGAGACACACCATGTTCAAACTG‑ATTAAGAAGTTAGGCCAACTGCTGGTTCGTATGTACAACGTGGAAGCCAAGCGACTGAACGATGAGGCTCGTAAAGAGGCCACACAGTCACGataatcaataggagaaatcactatgatcgtttctgacatcgaagctaacgccct                                                                                                                                                                                                                                                   >  1:25070‑M1/1‑247 (MQ=255)`  
                                                        `gaagaaGAGTCACACTCAGAGTCAACAGACTGGTCCAACGACACTGACTTCTGACAGGATTCTTGATGACTTTCCAGACGACTACGATAAGTTTCGCTGGAGAGTCCCATTCTAATACTACTCACTAAAGGAGACACACCATGTTCAAACTG‑ATTAAGAAGTTAGGCCAACTGCTGGTTCGTATGTACCACGTGGAAGTCAAGCGACTGAACGATGAGGCTCGTAAAGAGGCCACACAGTCACGataatcaataggagaaatcaatatgatcgtttctgacatcgaagctaacgccctctt                                                                                                                                                                                                                                                >  1:135902‑M1/1‑244 (MQ=255)`  
                                                          `gctcggagatgtgtataagagacaggactggtccaacgacaatgacttctgACAGGATTCTTGATGACTTTCCAGACGACTACGAGAAGTTTCGCTGGAGAGTCCCATTCTAATACGACTCACTAAAGGAGACACACCATGTTCAAACTG‑ATTAAGAAGTTAGGCCAACTGCTGGTTCGTATGTACAACGTGGAAGCCAAGCGACTGAACGATGAGGCTCGTAAAGAGGCCACACAGTCACGataatcaataggagaaatcaatatgatcgtttctgacatcgaagctaacgccctcttag                                                                                                                                                                                                                                              <  1:525547‑M1/276‑60 (MQ=255)`  
                                                            `aatagtcaAACTCAGAGTCAACAGATTGGTCCATCGACACTGACTTCTGACAGGTTTCGTGATGACTGTCCAGACGACTACGAGAAGTGTCGCTGGAGAGTCCCATTCTAATACGACTCACTAAAGGAGACACACCATGTTCAAACTG‑ATTAAGAAGTTAGGCCAACTGCTGGTTCGTATGTACAACGTGGAAGCCAAGTGACTGAACGATGAGGCTCGTAAAGAGGCCACACAGTCACGataatcaataggagaaatcaatatgatcgtttctgacatcgaagctaacgccctcttagag                                                                                                                                                                                                                                            <  1:148403‑M1/298‑62 (MQ=255)`  
                                                            `aagaGTCACACTCAGAGGCAACAGACTGGTCCAACGACACTGACTTCTGACAGGATTCTTGATGACTTGCCAGACGACTACGAGAAGTTTCGCTGGAGAGTCCCATTCTAATACGACTCACTAAAGGAGACACACCATGTTCAAACTG‑ATTAAGAAGTTAGGCCAACTGCTGGTTCGTATGTACAACGTGGAAGCCAAGCGACTGAACGATGAGGCTCGTAAAGAGGCCACACAGTCACGataatcaataggagaaatcaatatgatcgtttctgacatcgaagctaacgccctcttagag                                                                                                                                                                                                                                            <  1:18473‑M1/301‑62 (MQ=255)`  
                                                             `agatTCACACTCAGAGTCAACAGACTGGTCCAACGACACTGACTTCTGACAGGATTCTTGATGACTTTCCAGACTACTACGAGAAGTTTCTCTGGTGAGTCCCATTCTAATACTACTCATTAAAGGACACACACCATGTTCTAACTG‑TTTAAGAAGTTTTGCAAACTGCTGATTCGTATGTACCACGTGTAAGCAAAGCGACTGAACGATGAGTCTCGTAAAGAGTCCACACAGTCACGataatcaataggagaaatcaatatgatcgtttttgacatcgaagctaacgccatcttagttt                                                                                                                                                                                                                                           >  1:537936‑M1/1‑239 (MQ=255)`  
                                                              `gctcggagatgtgtataatgtacagggtccaatgacactgacttctgacaggATTCTTGATGACTTTCCAGACGACTACGAGAAGTTTCGCTGGAGAGTCCCATTCTAATACGACTCACTAAAGGAGACACACCATGTTCAAACTG‑ATTAAGAAGTTAGGCCAACTGCTGGTTCGTATGTACAACGTGGAAGCCAAGCGACTGAACGATGAGGCTCGTAAAGAGGCCACACAGTCACAataatcaataggagaaatcaatatgatcgtttctgacatcgaagctaacgccctcttagagag                                                                                                                                                                                                                                          <  1:582456‑M1/276‑64 (MQ=255)`  
                                                                `gTCACACTCAGAGTCAACAGACTTGTCCAACGACACTGACTTCTGACAGGATTCTTGATGACTTTCCAGACGACTACGAGAAGTTTCGCTGGAGAGTCCCATTCTAATACGACTCACTAAAGGAGACACACCATGTTCAAACTG‑ATTAAGAAGTTAGGCCAACTGCTGGTTCGTATGTACAACGTGGAAGCCAAGCGACTGAACGATGAGGCTCGTAAAGAGGCCACACAGTCACGataatcaataggagaaatcaatatgatcgtttctgacatcgaagctaacgccctcttagagagcg                                                                                                                                                                                                                                        <  1:462244‑M1/301‑66 (MQ=255)`  
                                                                `gTCACACTCAGAGTCAACAGACTGGTCCAACGACACTGACTTCTGACAGGATTCTTGATGACTTTCCAGACGACTACGAGAAGTTTCGCTGGAGAGTCCCATTCTAATACGACTCACTAAAGGAGACACACCATGTTCAAACTG‑ATTAAGAAGTTAGGCCAACTGCTGGTTCGTATGTACAACGTGGAAGCCAAGCGACTGAACGATGAGGCTCGTAAAGAGGCCACACAGTCACGataatcaataggagaactcaatatgatcgtttctgacatcgaagctaacgcactcttagagctgt                                                                                                                                                                                                                                        >  1:609129‑M1/1‑236 (MQ=255)`  
                                                                    `cacTCAGAGTCAACAGACTGGTCCAACGACACTGACTTCTGACAGGATTCTTGATGACTTTCCAGACGACTACGAGAAGTTTCGCTGGAGAGTCCCATTCTAATACGACTCACTAAAGGAGACACACCATGTTCAAACTG‑ATTAAGAAGTTAGGCCAACTGCTGGTTCGTATGTACAACGTGGAAGCCAAGCGACTGAACGATGAGGCTCGTAAAGAGGCCACACAGTCACGataatcaataggagaaatcaatatgatcgtttctgacatcgaagctaacgccctcttagagctgtctct                                                                                                                                                                                                                                    >  1:593180‑M1/1‑232 (MQ=255)`  
                                                                      `gatgtgtataagagacaggtccaacGACACTGACTTCTGACAGGATTCTTGATGACTTTCCAGACGACTACGAGAAGTTTCGCTGGAGAGTCCCATTCTAATACGACTCACTAAAGGAGACACACCATGTTCAAACTG‑ATTAAGAAGTTAGGCCAACTGCTGGTTCGTATGTACAACGTGGAAGCCAAGCGACTGAACGATGAGGCTCGTAAAGAGGCCACACAGTCACGataatcaataggagaaatcaatatgatcgtttctgacatcgaagctaacgccctcttagagagcgtcacta                                                                                                                                                                                                                                  <  1:730945‑M1/289‑72 (MQ=255)`  
                                                                      `gatgtgtataagagaaaggtccaacgacactgacttCTGACAGGATTCTTGATGACTTTCCAGACGACTACGAGAAGTTTCGCTGGAGAGTCCCATTCTAATACTACTCACTAAAGGAGACACACCATGTTCAAACTG‑ATTAAGAAGTTAGGCCAACTGCTGGTTCGTATGTACAACGTGGAAGCCAAGCGACTGAACGATGAGGCTCGTAAAGAGGCCACACAGTCACGataatcaataggagaaatcaatatgatcgtttctgacatcgaagctaacgccctcttagagagcatcacta                                                                                                                                                                                                                                  <  1:9626‑M1/284‑72 (MQ=255)`  
                                                                      `cTCAGAGTCAACAGACTGGTCCAACGACACTGACTTATGACAGGATTCTTGATGACTTTCCAGACGACTACGAGAAGTTTCGATGGAGAGTCCCATTCTAATACGACTCACTAAAGGAGACACACCATGTTCAAACTG‑ATTAAGAAGTTAGGCCAACTGCTGGTTCGTATGTACAACGTGGAAGCCAAGCGACTGAACGATGAGGCTCGTAAAGAGGCCACACAGTCACGataatcaataggagaaatcaatatgatcgtttctgacatcgaagctaacgccctcttagagagcgtcacta                                                                                                                                                                                                                                  <  1:285583‑M1/301‑72 (MQ=255)`  
                                                                      `cTCAGAGTCAACAGACTGGGCCAACGACACTGACTTCTGAAAGGATTCTTGATGACTTTCCAGACGACTACGAGAAGTGTCGCTGGAGAGTCCCATTCTAATACGACTCACTAAAGGAGACACACCATGTTCAAACTG‑ATTAAGAAGTTAGGCCAACTGCTGGTTCGTATGTACAACGTGGAAGCCAAGCGACTGAACGATGAGGCTCGTAAAGAGGCCACACAGTCACGataatcaataggagaaatcaatatgatcgtttctgacatcgaagctaacgccctcttagagagcgtcacta                                                                                                                                                                                                                                  <  1:812574‑M1/301‑72 (MQ=255)`  
                                                                              `cAACAGACTGGTCCAACGACACTGACTTCTGACAGGATTCTTGATGACTTTCCAGACGACTACGAGAAGTTTCGCTGGAGAGTCCCATTCTAATACTACTCACTAAAGGAGACACACCATGTTCAAACTG‑ATTAAGAAGTTAGGCCAACTGCTGGTTCGTATGTACAACGTGGAAGCCAAGCGACTGAACGATGAGGCTCGTAAAGAGGCCACACAGTCACGataatcaataggagaaatcaatatgatcgtttctgacatcgaagctaacgccctgtctcttatacacatctccgagccc                                                                                                                                                                                                                          >  1:728112‑M1/1‑222 (MQ=255)`  
                                                                              `cAACAGACTGGTCCAACGACACTGACTTCTGACAGGATTCTTGATGACTTTCCAGACGACTACGAGAAGTTTCGCTGGAGAGTCCCATTCTAATACGACTCACTAAAGGAGACACACCATGTTCAAACTG‑ATTAAGAAGTTAGGCCAACTGCTGGTTCGTATGTACAACGTGGAAGCCAAGCGACTGAACGATGAGGCTCGTAAAGAGGCCACACAGTCACGataatcaataggagaaatcaatatgatcgtttctgacatcgaagctaacgccctcttagagagcgtcactaagttccac                                                                                                                                                                                                                          <  1:106750‑M1/301‑80 (MQ=255)`  
                                                                              `cAACAGACTGGTCCAACGACACTGACTTCTGACAGGATTCTTGATGACTTTCCAGACGACTACGAGAAGTTTCGCTGGAGAGTCCCATTCTAATACGACTCACTAAAGGAGACACACCATGTTCAAACTG‑ATTAAGAAGTTAGGCAAACTGCTGGTTCGTATGTACAACGTGGAAGCCAAGCGACTGAGCGATGAGGCTCGTAAAGAGGCCACACAGTCACGataatcaataggagaaatcaatatgatcgtttctgacatcgaagctaacgccctcttagagctgtctcttatacacatt                                                                                                                                                                                                                          >  1:476419‑M1/1‑222 (MQ=255)`  
                                                                              `aagagacagggtccaacgacACTGACTTCTGACAGGATTCTTGATGACTTTCCAGACGACTACGAGAAGTTTCGCTGGAGAGTCCCATTCTAATACGACTCACTAAAGGAGACACACCATGTTCAAACTG‑ATTAAGAAGTTAGGCCAACTGCTGGTTCGTATGTACAACGTGGAAGCCAAGCGACTGAACGATGAGGCTCGTAAAGAGGCCACACAGTCACGataatcaataggagaaatcaatatgatcgtttctgacatcgaagctaacgccctcttagagagcgtcactaagttccac                                                                                                                                                                                                                          <  1:510746‑M1/292‑80 (MQ=255)`  
                                                                                `atggttataagagacagcactgacttctgaaaggattCTTGATGACTTTCCAGAAGACTACGAGAAGTTTAGCTGGAGAGTCACATTCTAATACGACTCACTAAAGGAGACACACCATGTTCAAACTG‑ATTAAGAAGTTAGGCCAACTGCTGGTTCGTATGTACAACGTGGAAGCCAAGCGACTGAACGATGAGGCTCGTAAAGAGGCCACACAGTCACGataatcaataggagaaatcaatatgatcgtttctgacatcgaagctaacgccctcttagagagcgtcactaagttccactg                                                                                                                                                                                                                        <  1:140857‑M1/284‑82 (MQ=255)`  
                                                                                `aCAGACTGGTCCAACGACACTGACTTCGGACAGGATTCTTGATGACTTTCCAGACGACTACGAGAAGTTTCGCTGGAGAGTCCCATTCTAATACGACTCACTAAAGGAGACACACCATGTTCAAACTG‑ATTAAGAAGTTAGGCCAACTGCTGGTTCGTATGTACAACGTGGAAGCCAAGCGACTGAACGATGAGGCTCGTAAAGAGGCCACACAGTCACGataatcaataggagaaatcaatatgatcgtttctgacatcgaagctaacgccctcttagagagcgtcactaagttccactg                                                                                                                                                                                                                        <  1:589186‑M1/301‑82 (MQ=255)`  
                                                                                `aCAGACGGGGCCAACGACACTGACTTCTGACAGGATTCTTGATGACTTGCAAGACGACTACGAGAAGTTTCGCTGGAGAGTCCCATTCTAATACGACTCACTAAAGGAGACACACCATGTTCAAACTG‑ATTAAAAAGTTAGGCCAACTGCTGGTTCGTATGTACAACGTGTAAGCCAAGCGACTGAACGATGAGGCTCGTAAAGAGGCCACACAGTCACGataagcaagaggagaaatcaatatgatcgtttctgacatcgaagctaacgccctcttagagagcgtcactaagttccactg                                                                                                                                                                                                                        <  1:343609‑M1/301‑82 (MQ=255)`  
                                                                                   `gACTCGTCCAACGACACTGACTTCTGACAGGATTCTTGATTACTTTCCAGACGACTACGAGAAGTTTCGCTGGAGAGTCCCATTCTAATACGACTCACTAAATGAGACACACCATGTTCAAACTG‑ATTAAGAAGTTAGGCCAACTGCTGGTTCGTATGTACAACGTGGAAGCCAAGCGACTGAACGATGAGGCTCGTAAAGAGGCCACACAGTCACGataatcaataggagaaatcaatatgatcgtttctgacatcgaagctaacgccctcttagagagcgtcactaagttccactgctg                                                                                                                                                                                                                     >  1:44624‑M1/1‑217 (MQ=255)`  
                                                                                     `cTGTTCCAACGACACTGACTTCTGACAGGATTCTTGATGACTTTCCAGACGACTACGAGAAGTTTCGATGGAGAGTACAATTCTAATACGACTCACTAAAGGAGACACACCATGTTCAAACTG‑ATTAAGAAGTTAGGCCAACTGCTGGTTCGTATGTACAACGTGGAAGCCAAGCGACTGAACGATGAGGCTCGTAAAGAGGCCACACAGTCACGataatcaataggagaaatcaatatgatcgtttctgacatcgaagctaacgccctcttagagagctgtctcttatacacatctccga                                                                                                                                                                                                                   >  1:330274‑M1/1‑215 (MQ=255)`  
                                                                                      `tGGTCCAACGACACTGACTTCTGACAGGATTCTTGATGACTTTCCAGACGACTACGAGAAGTTTCGCTGGAGAGTCCCATTCTAATACGACTCACTAAAGGAGACACACCATGTTCAAACTG‑ATTAAGAAGTTAGGCCAACTGCTGGTTCGTATGTACAACGTGGAAGCCAAGCGACTGAACGATGAGGCTCGTAAAGAGGCCACACAGTCACGataatcaataggagaaatcaatatgatcgtttctgacatcgaagctaacgccctcttagagagcgtcactaagttccactgcggggt                                                                                                                                                                                                                  <  1:370755‑M1/301‑88 (MQ=255)`  
                                                                                       `ggTCCAACGACACTGACTTCTGACAGGATTCTTGATGACTTTCCAGACGACTACGAGAAGTTTCGCTGGAGAGTCCCATTCTAATACGACTCACTAAAGGAGACACACCATGTTCAAACTG‑ATTAAGAAGTTAGGCCAACTGCTGGTTAGTATGTACAACGTGGAAGCCAAGCGACTGAACGATGAGGCTCGTAAAGAGGCCACACAGTCACGataatcaataggagaaatcaatatgatcgtttctgacatcgaagctaacgccctcttagagagcgtcactaagttccactgcggggtt                                                                                                                                                                                                                 >  1:554982‑M1/1‑213 (MQ=255)`  
                                                                                       `ggTCCAACGACACTGACTTCTGACAGGATTCTTGATGACTTTCCAGACGACTACGAGAAGTGTCGCTGGAGAGTCCCCTTCTAATACGACTCAAGAAAGGAGACACACAATGATCAAACTG‑ATTAAGACGTTAGGCCAACTGCTGGTTAGTATGTCCACCGTGGAAGCCAAGCGACTGACCGATGAGGCTCGTAAAGAGGAGACACAGTCACGataatcaataggagcaatcaatatgatcgtttctgacatcgaagctaacgctctgttagcgtgcgtctctacgttccaatgcttggtt                                                                                                                                                                                                                 >  1:489594‑M1/1‑213 (MQ=255)`  
                                                                                        `gTCCAACGACACTGACTTCTGACAGGATTCTTGATGACTTTCCAGACGACTACAAGAAGTTTCGCTGGAGAGTCCCATTCTAATACGACTCACTAAAGGAGACACACCATGTTCAAACTG‑ATTAAGAAGTTAGGCCAACTGCTGGTTCGTATGTACAACGTGGAAGCCAAGCGACTGAACGATGAGGCTCGTAAAGAGGCCACACAGTCACGataatcaataggagaaatcaatatgatcgtttctgacatcgaagctaacgacctcttagagagcgtcactaagttcaactgcggggtta                                                                                                                                                                                                                >  1:805703‑M1/1‑212 (MQ=255)`  
                                                                                           `cAACGACACTGACTTCTGACAGGATTCTTGATGACTTTCCAGACGACTACTAGAAGTTTCGCTGGAGAGTCCCATTCTAATACGACTCACTAAAGGAGACACACCATGTTCAAACTG‑ATTAAGAAGTTAGGCCAACTGCTGGTTCGTATGTACAACGTGGAAGCCAAGCGACTGAACGATGAGGCTCGTAAAGAGGCCACACAGTCACGataatcaataggagaaatcaatatgatcgtttctgacatcgaagctaacgccctcttagagagcgtcactaatttccaccgcggggttatct                                                                                                                                                                                                             >  1:737757‑M1/1‑209 (MQ=255)`  
                                                                                                `acacTGACTTCTGACAGGATTCTTGATGACTTTCAAGACGACTACGAGAAGTTTCGCTGGAGAGTCCCATTCTAATACGACTCACTAAAGGAGACACACCATGTTCAAACTG‑ATTAAGAAGTTAGGCCAACTGCTGGTTCGTATGTACAACGTGGAAGCCAAGCGACTGAACGATGAGGCTAGTAAAGAGGCCACACAGTCACGataatcaataggagaaatcaatatgatcgtttctgacatcgaagctaacgccctcttagagagcgtcactaagttccactgcggggttatctacgac                                                                                                                                                                                                        >  1:782325‑M1/1‑204 (MQ=255)`  
                                                                                                     `gACTTCTGACAGGATTCTTGATGACTTTCCAGACGACTACGAGAAGTTTCGCTGGAGAGTCCCATTCTAATACGACTCACTAAAGGAGACACACCATGTTCAAACTG‑ATTAAGAAGTTAGGCCAACTGCTGGTTCGTATGTACAACGTGGAAGCCAAGCGACTGAACGATGAGGCTCGTAAAGAGGCCACACAGTCACGataatcaataggagaaatcaatatgatcgtttctgacatcgaagctaacgccctcttagagagcgtcactaagttccactgcggggttatctacgactactc                                                                                                                                                                                                   <  1:434639‑M1/301‑103 (MQ=255)`  
                                                                                                     `gACTTCTGACAGGATTCTTGATGACTTTCCAGACGACTACGAGAAGTTTCGCTGGAGAGTCCCATTCTAATACGACTCACTAAAGGAGACACACCATGTTCAAACTG‑ATTAAGAAGTTAGGCCAACTGCTGGTTCGTATGTACAACGTGGAAGCCAAGCGACTGAACGATGAGGATCGTAAAGAGGCCACACAGTCACGataatcaataggagaaatcaatatgatcgtttctgacatcgaagctaacgccctcttagagagcgtcactaagttccacctgtctcttatacacatctccta                                                                                                                                                                                                   >  1:214389‑M1/1‑199 (MQ=255)`  
                                                                                                     `gACTTCTGACAGGATTCTTGATGACTTTCCAGACGACTACGAGAAGTTTCGCTGGAGAGTCCCATTCTAATACGACTCACTAAAGGAGACACACCATGTTCAAACTG‑ATTAAGAAG‑TAGGCCAACTGCTGGTTCGTATGTACAACGTGGAAGCCAAGCGACTGAACGATGAGGCTCGTAAAGAGGCCACAAAGTCACGataatcaataggagaaatcaatatgatcatttctgacctcgaagctaacgccctcttaaagagcgtccctaagttccactgcgggattatctcagactactcc                                                                                                                                                                                                  >  1:696410‑M1/1‑198 (MQ=255)`  
                                                                                                      `aCTTCTGACAGGATTCTTGATTACTTTCCAGACGACTACGAGAAGTTTCGCTGGAGAGTCCAATTCTAATACGACTCACTAAAGGAGACACAGCATGTTCAAACTG‑ATTAAGAAGTTAGGCCAACTGCTGGTTCGTATGTACAACGTGGAAGCCAAGCGACTGAACGATGAGGCTCGTAAAGAGGCCACACAGTCACGataatcaataggagaaatcaatataatcgtttctgacatcgaagctaatgccctcttagagagagtcactaagttccactccgtggttatttacgactattcc                                                                                                                                                                                                  >  1:500553‑M1/1‑198 (MQ=255)`  
                                                                                                           `tGAAAGGATTCTTGATGACTTTCCAGACGACTACTATAAGTTTCGCTGGAGAGTCCCATTCTAATACGACTCACTAAAGGAGACACACCATGTTCAAACTG‑ATTAAGAAGTTAGGCCAACTGCTGGTTCGTATGTACAACGTGGAAGCCAAGCGACTGAACGATGAGGCTCGTAAAGAGGCCACACAGTCACGataatcaataggagaaatcaatatgatcgtttctgacatcgaagctaacgccctcttagagagcgtcactaagttccacttcggggttatctacgactactccaccgc                                                                                                                                                                                             <  1:514724‑M1/301‑109 (MQ=255)`  
                                                                                                               `aGGATTCTTGATGACTTTCCAGACGACTACGAGAAGTTTCGCTGGAGAGTCCCATTCTAATACGACTCACTAAAGTAGACACACCATGTTCAAACTG‑ATTAAGAAGTTAGGCCAACTGCTGGTTCGTATGTACAACGTGGAAGCCAAGCGACTGAACGATGAGGCTCGTAAAGAGGCCACACAGTCACGataatcaataggagaaatcaatatgatcgtttctgacatcgaagctaacgccctcttagagagcgtcactaagttccactgcggggttatctacgactactccaccactgag                                                                                                                                                                                         <  1:615160‑M1/301‑113 (MQ=255)`  
                                                                                                                   `ttCTTGATGACTTTCCAGACTACTACGAGAAGTTTCGCTGGAGAGTCCCATTCTAATACGACTCACTAAAGGAGACACACCATGTTCAAACTG‑ATTAAGAAGTTAGGCCAACTGCTGGTTCGTATGTACAACGTGGAAGCCAAGCGACTGAACGATGAGGCTCGTAAAGAGGCCACACAGTCACGataatcaataggagaaatcaatatgatcgtttctgacatcgaagctaacgccctcttagagagcgtcactaagttacactgcggggttatctacgactactccaccgctgagtacg                                                                                                                                                                                     <  1:117137‑M1/301‑117 (MQ=255)`  
                                                                                                                   `ttCTTGATGACTTTCCAGACGACTACGAGAAGTTTCGCTGGAGAGTCCCATTCTAATACGACTCACTAAAGGAGACACACCATGTTCAAACTG‑ATTAAGAAGTTAGGCCAACTGCTGGTTCGTATGTACAACGTGGAAGCCAAGCGACTGAACGATGAGGCTCGTAAAGAGGCCACACAGTCACGataatcaataggagaaatcaatatgatcgtttctgacatcgaagctaacgccctcttagagagcgtcactaagttccactgcggggttatctacgctgtctcttatacacatctcc                                                                                                                                                                                     >  1:637741‑M1/1‑185 (MQ=255)`  
                                                                                                                     `cTTGATGACTTTCCAGACGACTACGAGAAGTTTCGCTGGAGAGTCCCATTCTAATACGACTCACTAAAGGAGACACACCATGTTCAAACTG‑ATTAAGAAGTTAGGCCAACTGCTGGTTCGTATGTACAACGTGGAAGCCAAGCGACTGAACGATGAGGCTCGTAAAGAGGCCACACAGTCACGataatcaataggagaaatcaatatgatcgtttctgacatcgaagctaacgccctcttagagagcgtcactaagttccactgcggggttatctactgtctcttatacacatctccgagc                                                                                                                                                                                   >  1:237882‑M1/1‑183 (MQ=255)`  
                                                                                                                        `ggtacggagatgtgtataagagacagggttcgctggaaattccctgtctaatacgactCACTAAAGGAGACACACCATGTTCAAACTG‑ATTAAGAAGTTAGGCCAACTGCTGGTTCGTATGTACAACGTGGAAGCAAAGCGACTGAACGATGAGGCTCGTAAAGAGGCCACACAGTCAAGataatcaataggagaaataaatatgatcgtttctgacatcgaagctaacgccctcttagagagcgtcactaagttccactgcggggttatctacgactactccaccgctgagtacataagc                                                                                                                                                                                <  1:150402‑M1/273‑122 (MQ=255)`  
                                                                                                                             `agttccAGATGACTACGAGAAGTTTCGCTGGAGAGTCCCATTCTAATACGTCTCACTAAAGGAGACACACCATGTTCAAACTG‑ATTAAGAAGTTAGGCCAACTGCTGGTTCGTATGTACAACGTGGAAGCCAAGCGACTGAACGATGAGGCTCGTAAAGAGGCCACACAGTCACGataatcaataggagaaatctatatgatcgtttctgacatcgaagctaacgccctcttagagagcgtcactaagttccactgcggggttatctacgactactccaccgctgagtacgtaagctaccg                                                                                                                                                                           <  1:205575‑M1/299‑127 (MQ=255)`  
                                                                                                                                   `aGACGACTACGAGAAGTTTCGCTGGAGAGTCCCATTCTAATACGACTCACTAAAGGAGACACACCATGTTCAAACTG‑ATTAAGAAGTTAGGCCAACTGCTGGTTCGTATGTACAACGTGGAAGCCAAGCGACTGAACGATGAGGCTCGTCAAGAGGCCACACAGTCACGataatcaataggagaaatcactatgatcgtttctgacatcgaagctaacgccctcttagagagcgtcactaagttcccctgcggggttatctacgactactcccccgctgagtacgtaagctacctgtctct                                                                                                                                                                     >  1:382399‑M1/1‑169 (MQ=255)`  
                                                                                                                                                    `ttCGCTTGAGAGTCCCATTCGAATACGACTCACTAAATGATACACACCATGTTCAAACTG‑ATTAAGAAGTTAGGCCAACTGCTGGTTCGTATGTACAACGTGGAAGCCAAGCGACTGAACGATGAGGCTCGTAAAGATGCCACACAGTCACGataatcaataggagaaatcaatatgatcgtttctgacatcgaagctaacgccctcttatagagcgtcactaagttccactgcggggttatctacgactactccaccgctgagtacgtaagctaccgtccgagtgacttcggtgcgtatc                                                                                                                                                    <  1:396945‑M1/301‑150 (MQ=255)`  
                                                                                                                                                    `ttCGCTGGAGAGTCCCATTCTAATACGACTCACTAAAGGAGACACACCATGTTCAAACTG‑ATTAAGAAGTTAGGCCAACTGCTGGTTCGTATGTACAACGTGGAAGCCAAGCGACTGAACGATGAGGCTCGTAAAGAGGCCACACAGTCACGataatcaataggagaaatcaatatgatcgtttctgacatcgaagctaacgccatcttagagagcgtcactacgttccactgcggggttatctacgactactacaccgctgagtacgtcagctaccgtccgagtgacttcggtgcgtttc                                                                                                                                                    >  1:427262‑M1/1‑152 (MQ=255)`  
                                                                                                                                                        `gtgGAGAGTCCCAATCTAAAACGAACCACTAAAGGAGGCAAACCCTGGTCCAAATGAATTAGAAAGTTAGGCC‑ACTGGTTGTTCGTATGTACAACGTGGAAGCCAAGCGACTGAACGATGAGGCTCGTAAAGAGGCCACACAGTCACGataatcaataggagaaatcaatatgatcgtttctgacatcgaagctaacgccatcttagagagcgtcactaagttccactgcggggttatctacgactactccaccgctgagtacgtaagctaccgtccgagtgacttcggtgcgtatctgga                                                                                                                                                <  1:157366‑M1/300‑154 (MQ=255)`  
                                                                                                                                                          `ggAGAGTCCCATTCTAATACGACTCACTAAAGGAGACACACCATGTTCAAACTG‑ATTAAGAAGTTAGGCCAACTGCTGGTTCGTATGTACAACGTGGAAGCCAAGCGACTGAACGATAAGGCTCGTAAAGAGGCCACACAGTCACGataatcaataggagaaatcaatatgatcgtttctgacatcgaagctaaagccctcttagagagcgtcactaagttccactgcggggttatctacgactactccaccgctgagtacgtaagctaccgtccgagtgacttcggtgctgtctcttata                                                                                                                                              >  1:498714‑M1/1‑146 (MQ=255)`  
                                                                                                                                                           `gagaGTCCCATTCTAATACGACTCACTAAAGGAGACACACCATGTTCAAACTG‑ATTAAGAAGTTAGGCCAACTGCTGGTTCGTATGTACAACGTGGAAGCCAAGCGACTGAACGATGAGGCTCGTAAAGAGGCCACACAGTCACGataatcaataggagaaatcaatatgatcgtttctgacatcgaagctaacgccctcttagagagcgtcactaagttccactgcggggttatctacgactactccaccgctgagtacgtaagctaccgtccgagtgacttcggtgcgtatctggatgc                                                                                                                                             <  1:353653‑M1/301‑157 (MQ=255)`  
                                                                                                                                                               `gTCCCATTCTAATACGACTCACTAAAGGAGACACACCATGTTCAAACTG‑ATTAAGAAGTTAGGCCAACTGCTGGTTCGTATGTACAACGTGGAAGCCAAGCGACTGAACGATGAGGCTCGTAAAGAGGCCACACAGTCACGataatcaataggagaaatcaatatgatcgtttctgacatcgaagctaccgccctcttcgagagcttcactaagttccactgcgtggttatctacgactactccaccgctgcgtacgtaagctaccgtccgagtgacttcggtgcgtatctggatgcgcct                                                                                                                                         >  1:298454‑M1/1‑141 (MQ=255)`  
                                                                                                                                                                   `cATTCTAATACGACTCACTAAAGGTGACACACCATGTTCAAACTG‑ATTAAGAAGTTAGGCCAACTGCTGGTTCGTATGTACAACGTGGAAGCCAAGCGACTGAACGATGAGGCTCGTAAAGAGGCCACACAGTCACGataatcaataggagaaatcaatatgatcgtttctgacatcgaagctaacgacatcttagagagcgtcactaagttccactgcggggttatctacgactactccaccgctgagtacgtaagctaccgtccgagtgacttcggtgcgtatctggctgcgctggaag                                                                                                                                     >  1:739757‑M1/1‑137 (MQ=255)`  
                                                                                                                                                                     `ttCTAATACGACTCACTAAAGGAGACACACCATGTTCAAACTG‑ATTAAGAAGTTAGGCCAACTGCTGGTTCGTATGTACAACGTGGAAGCCAAGCGACTGAACGATGAGGCTCGTAAAGAGGCCACACAGTCACGataatcaataggagaaatcaatatgatcgtttctgacatcgaagctaacgccctcttatagagcgtcactaagttccactgcggggttatctacgactactccaccgctgagtacgtaagctaccgtccgagtgacttcggtgcgtatctggatgcgctggaagcc                                                                                                                                   >  1:25534‑M1/1‑135 (MQ=255)`  
                                                                                                                                                                     `ttCTAATACGACTCACTAAAGGAGACACACCATGTTCAAACTG‑ATTAAGAAGTTAGGCCAACTGCTGGTTCG‑ATGTACAACGTGGAAGCCAAGCGACTGAACGATGAGGCTCGTAAAGAGGCCACACAGTCACGataatcaataggagaaatcaatatgatcgtttctgacatcgaagctaacgccctcttagagagcgtcactaagttccactgcggggttatctacgactactccaccgctgagtacgtaagctaccgtccgagtgacttaggtgcgtagctggatgcgctggaagccg                                                                                                                                  >  1:254275‑M1/1‑134 (MQ=255)`  
                                                                                                                                                                                         `ggAGACACACCATGTTCAAACTG‑ATTAAGAAGTTAGGCCAACTGCTGGTTCGTATGTACAACGTGGAAGCCAAGCGACTGAACGATGAGGTTCGTAAAGAGGCCACACAGTCACGctaatcaataggagaagtcaatatgatcgtttctgacatcgaagctaacgccatcttagagagcgtcactaagttccactgcggggttatctacgactactccaccgctgcttacgtaagctaccgtccgagtgacttcggtgcgtatctggatgcgctggaagccgaggttgcacgaggcggtct                                                                                                               >  1:704880‑M1/1‑115 (MQ=255)`  
                                                                                                                                                                                            `gaCACACCCTGTTCAAACTG‑ATTAAGAAGTTAGGCCAACTGCTGGTTCGTATGTACAACGTGGAAGCCAAGCGACTGAACGATGAGGCTCGTAAAGATGCCACACAGTCACGataatcaatagtagaaatcaatatgctcgtttctgacatcgaagctaacgccctcttagagagcgtcactaagttccactgcggggttatctacgactactccaccgctgagtacgtaagctaccgtccgagtgacttcggtgcgtatctggatgcgctggaagccgaggttgcacgaggcggtcttat                                                                                                            >  1:320253‑M1/1‑112 (MQ=255)`  
                                                                                                                                                                                              `cacacccgtttaaaactg‑attaagaagTTAGGCCAACTGCTGGTTCGTATGTACAACGTGGAAGCCAAGCGACTGAACGATGAGGCGCGTAAAGAGGCCACACAGTCACGataatcaataggagaaatcaatatgatcgtttctgacatcgaagctaacgccctctgagagagcgtcactaagttccactgcggggttatctacgactactccaccgctgagtacgtaagctaccgtccgagtgacttcggtgcgtatctggatgcgctggaagccgaggttgcacgaggcggtcttattg                                                                                                          <  1:386830‑M1/289‑192 (MQ=255)`  
                                                                                                                                                                                              `cacacCATGTTCAAACTG‑ATTAAGAAGTTAGGCCAACTGCTGGTTCGTATGTACAACGTGGAAGCCAATCGACTGAACGATGAGGCTCGTAAAGAGGCCACACAGTCACGataatcaataggagaaatcaatatgatcgtttctgacatcgaagctaacgccctcttagagagcgtcactaagttccactgcggggttatctacgactactccaccgctgagtacgtaagctaccgtccgagtgacttcggtgcgtatctggatgcgctggaagccgaggttgcacgaggcggtcttattg                                                                                                          <  1:562514‑M1/301‑192 (MQ=255)`  
                                                                                                                                                                                              `aacaacatGTTAAAACTG‑ATTAAGAAGTTAGGCCAACTGCTGGTTCGTATGTACAACGTGGAAGCCAAGCGACTGAACGATGAGGCTCGTAAAGAGGCCACACAGTCACAataatcaataggagaaatcaatatgatcgtttctgacatcgaagctaacgccctcttagagagcgtcactaagttccactgcggggttatctacgactactccaccgctgagtacgtaagctaccgtccgagtgacttcggtgcgtatctggatgcgctggaagccgaggttgcacgaggcggtcttattg                                                                                                          <  1:543872‑M1/300‑192 (MQ=255)`  
                                                                                                                                                                                                                 `aTTAAGAAGTTAGGCCAACTGCTGGTTAGTATGAAAAACGTGGAAGCCAAGCGACTGAACGATGAGGCTCGTAAAGAGGCCACACAGTCACGataatcaataggagaaatcaatatgatcgtttccgacatcgaagctaatgccctcttagagagcgtcactaagttccactgcggggttatctacgactactccaccgctgagtacgtaagctaccgtccgagtgacttcggtgcgtatcttgatgcgctggaagccgaggttgcacgaggcggtcttattgtgttccacaacggtcaca                                                                                        <  1:623143‑M1/301‑210 (MQ=255)`  
                                                                                                                                                                                                                        `agTTAGGCCAACTGCTGGTTCGTATGTACAACGTGGAAGCCAAGCGACTGAACGATGAGGCTCGTAAAGAGGCCACACAGTCACGataatcaataggagaaatcaatatgatcgtttctgacatcgaagctaacgccctcttagagagcgtcactaagttccactgctgggttatctacgactactccaccgctgagtacgtaagctaccgtccgagtgacttcggtgcgtatctggatgcgctggaagccgaggttgcacgaggcggtcttattgtgttccacctgtctcttatacacat                                                                                 >  1:444126‑M1/1‑85 (MQ=255)`  
                                                                                                                                                                                                                        `agTTAGGCCAACTGCTGGTTCGTATGTACAACGTGGAAGCCAAGCGACTGAACGATGAGGCTCGTAAAGAGGCCACACAGTCACGataatcaataggagaaatcaatatgatcgtttctgacatcgaagctaacgccctcttagagagcgtcactaagttccactgcggggttatctacgactactccaccgctgagtacgtaagctaccgtccgagtgacttcggtgcgtatctggatgcgctggaagccgaggttgcacgaggcggtcttattgtgttccacaacggtcacaagtatga                                                                                 <  1:574124‑M1/301‑217 (MQ=255)`  
                                                                                                                                                                                                                                `aaacTGCTGGTTCGTATGTACAACGTGGAAGCCAAGCTACTGAACGATGAGGCTCGTAAAGAGGCCACACAGTCACGataatcaataggagaaatcaatatgatcgtttctgacatcgaagctaacgccctcttagagagcgtcactaagttcaactgcggggttatctacgactactccaccgctgagtacgtaagctaccgtccgagtgacttcggtgtgtatctggatgcgctggaagccgaggttgcacgaggcggtcttattgtgttccacaacggtaacaagtatgacgttcctg                                                                         <  1:655172‑M1/300‑225 (MQ=255)`  
                                                                                                                                                                                                                                  `aCTGCGGGTTCGTATGTAAAACGTGGAAGCCAAGCGACTAAACGATGAGGCTCGTAAAGAGGCCACACAGTCACGataatcaataggagaaatcaatatggtcgtttctgacatcgaagctaacgccctcttagagagcgtcactaagttccactgcggggttatctacgactactccaccgccgagtacgtaagctaccgtccgagtgacttaggtgcgtatctggatgcgctggaagccgaggttgcacgaggcggtcttattgtgttccacaacggtcacaagtatgacgttcctgca                                                                       <  1:110851‑M1/301‑227 (MQ=255)`  
                                                                                                                                                                                                                                   `ctgctgGTTCGTATGTACAACGTGGAAGCCAAGCGACTGAACGATGAGGCTCGTAAAGAGGCCACACAGTCACGataatcaataggagaaatcaatatgatcgtttctgacatcgaagctaacgccctcttagagagcgtcactaagttccactgcggggttatctatgactactccaccgctgagtacgtaagctaccgtccgagtgacttcggtgcgtatctggatgcgctggaagccgaggttgcacgaggcggtcttattgtgttccacaacggtcacaagtatgacgatcctgcat                                                                      >  1:790966‑M1/1‑74 (MQ=255)`  
                                                                                                                                                                                                                                         `gTTCGTATGTACAACGTGGAAGCCAAGTGACTGAACGATGAGGCTCGTAAAGAGGCCACACAGTCACGataatcaataggagaaatcaatatgatcgtttctgacatcgaagctaacgccctcttagagagcgtcactaagttccactgcggggttatctactactactccaccgctgagtacgtaagctaccgtccgagtgacttcggtgcgtatctggatgcgctggaagccgaggttgcacgaggcggtcttattgtgttccacaacggtcacactgtctcttatacacaactccgag                                                                >  1:193098‑M1/1‑68 (MQ=255)`  
                                                                                                                                                                                                                                            `cGTATGTACAACGTGGAAGCCAAGCGACTGAACGATGAGGCTCGTAAAGAGGCCACACAGTCACGataatcaataggagaaatcaatatgatcgtttctgacatcgaagctaacgcactcttagagagcgtcactaagttccactgcggggttatctacgactactccaccgctgagtacgtaagctaccgtccgagtgacttcggtgcgtatctggatgcgctggaagccgaggttgcacgaggcggtcttattgtgttccacaacggtcacaagtatgacgttcctgcattgaccaaac                                                             >  1:258382‑M1/1‑65 (MQ=255)`  
                                                                                                                                                                                                                                                 `gTACAACGTGGAAGCCAAGCGACTGAACGATGAGGCTCGTAAAGAGGCCACACAGTCACGataatcaataggagaaatcaatatcatcgtttctgacatcgaagctaacgccctcttagagagcgtcactaagttccactgcggggttatctacgactactccaccgctgagtacgtaagctaccgtccgagtgacttcggtgcgtatctggatgcgctggaagccgaggttgcacgaggcggtcttattgtgttccacaacggtcacaagtatgacgctgtctcttatacacatctccga                                                        >  1:680542‑M1/1‑60 (MQ=255)`  
                                                                                                                                                                                                                                                       `cGTGGAAGCCAAGCTACTGAACGATGAGGCTCGTAAAGAGGCCACACAGTCACGataatcaataggagaaatcaatatgatcgtttctgacatcgaagctaacgccctcttagagagcgtcactaagttccactgcggggttatctacgactactccaccgctgagtacgtaagctaccgtccgagtgacttcggtgcgtatctggatgcgctggaagccgaggttgcacgaggcggtcttattgtgttccacaacggtcacaagtatcacgttcctgcattgaccaaactggcaaagttg                                                  <  1:325492‑M1/301‑248 (MQ=255)`  
                                                                                                                                                                                                                                                             `aGCCAAGCGACTGAACGATGAGGCTCGTAAAGAGGCCACACAGTAACGataatcaataggagaaatcaatatgatcgtttctgacatcgaagctaacgccctcttagagagcgtcactaagttccactgcggggttatctacgactactccaccgctgagtacgtaagctaccgtccgagtgacttcggtgcgtatctggatgcgctggaagccgaggttgcacgaggcggtcttattgtgttccacaacggtcacaagtatgacgttcctgcattgaccaaactggcaaagttgcaattg                                            <  1:707803‑M1/301‑254 (MQ=255)`  
                                                                                                                                                                                                                                                              `gCCAAGCGACTGAACGATGAGGCTCGCAAAGAGGCCACACAGTCACGataatcaataggagaaatcaatatgatcgtttctgacatcgaagctaacgccctcttagagagcgtcactaagttccactgcggggttatctacgactactccaccgctgagtacgtaagctaccgtccgagttacttcggtgcgtatctggatgcgctggaagccgaggttgcacgaggcggtcttattgtgttccacaacggtcacaagtatgacgttcctgcattgaccaaactggcaaagttgcaattga                                           >  1:230587‑M1/1‑47 (MQ=255)`  
                                                                                                                                                                                                                                                                `aaagCGACTGAACGATGGGTCTCGTAAAGAGGCCACACAGTCACGataatcaataggagaaatcaatatgatcgtttctgacatcgaagctaacgccctcttagagagcgtcactaagttccactgcggggttatctacgactactccaccgctgagtacgtaagctaccgtccgagtgacttcggtgcgtatctggatgcgctggaagccgaggttgcacgaggcggtcttattgtgttccacaacggtcacaagtatgacgttcctgcattgaccaaactggcaaagttgcaattgaac                                         <  1:175844‑M1/300‑257 (MQ=255)`  
                                                                                                                                                                                                                                                                  `aGCGACTGAACGATGAGGCTCGTAAAGAGGCCACACAGTCACGataatcaatagtagaaatcaatatgatcgtttatgacatcaaagctaacgcactcttagagagcgtcacttagttacactgcggggttatctacgactactccaccgctgagcacgtaagctaccgtccgagtgacgtcggtgcgtatctggatgcgctggaagccgagggtgcacgaggcggccttattgtcttcaccaacggtcacaagtatgacgttgctactttgaccaaactggcacagttgcaattgaaacg                                       >  1:171532‑M1/1‑43 (MQ=255)`  
                                                                                                                                                                                                                                                                   `gCGACTGAACGATGAGGCTCGTAAAGAGGCCACACAGTCACGataatcaataggagaaatcaatatgatcgtttctgacatcgaagctaacgccctcttagagagcgtcactaagttccactgcggggttatctacgactactccaccgctgagtacgtaagttaccgtccgagtgacttcggtgcgtatctggatgcgctggaagccgaggttgcacgaggcggtcttattgtgttccacaacgttcacaagtatgacgttcctgcattgaccaaactggcaaatttgcaattgaaccga                                      >  1:752373‑M1/1‑42 (MQ=255)`  
                                                                                                                                                                                                                                                                   `gCGACTGAACGATGAGGCTCGTAAAGAGGCCACACAGTCACGataatcaataggagaaatcaatatgatcgtttctgacatcgaagctaacgccctcttagagagcgtcactaagttccactgcggggttatctacgactactccaccgctgagtacgtaagctaccgtccgagtgacttcggtgcgtatctggatgcgctggaagccgaggttgcacgaggcggtcttattgtgttccacaacggtcacaagtatgacgttcctgcattgaccaaactctgtctcttatacacatctccc                                      >  1:409391‑M1/1‑42 (MQ=255)`  
                                                                                                                                                                                                                                                                    `cGACTGAACGATGAGGCTCGTAAAGAGGCCACACAGTCACGataatcaataggagaaatcaatatgatcgtttctgacatcgaagctaacgccctcttagagagcgtcactaagttccactgcggggttatctacgactactccaccgctgagtacgtaagctaccgtccgagtgacttcggtgcgtatctggatgcgctggaagccgaggttgcacgaggcggtcttattgtgttccacaacggtcacaagtatgacgttcctgcattgacccaactggcaaagttgcaattgaaccgag                                     >  1:268580‑M1/1‑41 (MQ=255)`  
                                                                                                                                                                                                                                                                     `aacTGAACGATGAGGCTCGTAAAGAGGCCACACAGTCACGataatcaataggagaaatcaatatgatcgtttctgacatcgaagctcactccctcttagagagcgtcactaagttccactgcggtgttatctacgactactacaccgctgagtacgtaagctaccgtcctagtgacttcggtgcgtatctggatgcgctggaagccgaggttgcacgaggcggtcttattgtgttccacaacggtcacaagtatgacgttcctgcattgaccaaactggcaaagttgcaattgaaccgaga                                    <  1:723086‑M1/300‑262 (MQ=255)`  
                                                                                                                                                                                                                                                                      `aCTGAACGATGAGGCTCGTAAAGAGGCCACACAGTCACGataatcaataggagaaatcaatatgatcgtttctgacatcgaagctaacgccctcttagagagcgtcactaagttccactgcggggttatctacgactactacaccgctgagtacgtaagctaccgtccgagtgacttcggtgcgtatctggatgcgctggaagcagaggttgcacgaggcggtcttattgtgttccacaacggtcacaagtatgacgttcctgcattgaccaaactggcaaagttgcaattgaaccgagag                                   <  1:41876‑M1/301‑263 (MQ=255)`  
                                                                                                                                                                                                                                                                                           `agagGCCACACAGTCACGataatcaataggagaaatcaatatgatcgtttctgacatcgaagctaacgccctcttagagagcgtcactaagttccactgcggggttatctacgactactccaccgctgagtacgtaagctaccgtccgagtgacttcggtgcgtatctggatgcgctggaagccgaggttgcacgaggcggtcttattgtgttccacaacggtcacaagtatgacgttcctgcattgaccaaactggcaaagttgcaattgaaccgagagttccaccttcctcgtgagaac              <  1:263021‑M1/301‑284 (MQ=255)`  
                                                                                                                                                                                                                                                                                              `ggCCACACAGTCACGataatcaataggagaaatcaatatgatcgtttctgacatcgaagctaacgccctcttagagagcgtcactaagttccactgtggggttatctacgactactccaccgctgagtacgtaagctaccgtccgagtgacttcggtgcgtatctggatgcgctggaagccgaggttgcacgaggcggtcttattgtgttccacaacggtcacaagtatgacgttcctgcattgaccaaactggcaaagttgcaattgaaccgagagttccaccttcctcgtgactgtccc           >  1:747704‑M1/1‑15 (MQ=255)`  
                                                                                                                                                                                                                                                                                                     `caGTCACGataatcaataggagaaagcaatatgatcgtttctgacatcgaacctaacgccctctgagagagcgtcactaagttccactgcggggttatctacgactactccaccgctgagtacgtaagctaccgtccgagtgacttcggtgcgtatctggatgcgctggaagccgaggttgcacgaggcggtcttattgtgttccacaacggtcacaagtatgacgttcctgcattgaccaaactggcaaagttgcaattgaaccgagagttccaccttcctcgtgagaactgtattgaca    <  1:799164‑M1/301‑294 (MQ=255)`  
                                                                                                                                                                                                                                                                                                     `caGTCACAataatcaataggagaaatcaatatgatcgtttctgacatcgaagctaacgccatcttagagagcgtcactaagttccacggcggggttatctacgactactccaccgatgagtacgtaagctaccgtccgagtgacttcggtgcgtatctggatgcgctggaagccgaggttgcacgaggcggtcttattgtgttcaacaacggtcacaagtatgacgttcccgcattgaccaaactggcaaagttgcaattgaaccgagagttccaccttcctcgtgagaactgtattgaca    <  1:302549‑M1/301‑294 (MQ=255)`  
                                                                                                                                                                                                                                                                                                      `aGTCACGataatcaataggagaaatcaatatgatcgtttctgacatcgaagctaacgccctcttagagagcgtcactaagttccactgcggggttatctacgactactccaccgctgagtacgtaagctaccgtccgagtgacttcggtgcgtctctggatgcgctggaagccgaggttgcacgaggcggtcttattgtgttccacaacggtcacaagtatgacgttcctgcattgaccaaactggcaaagttgcaattgaaccgagagttccaccttcctcgtgagaactgtattgacac   >  1:36621‑M1/1‑7 (MQ=255)`  
                                                                                                                                                                                                                                                                                                       `gTCATGataatcaataggagaaatcaatatgatcatttctgacatcgaagctaacgccctcttagagagcgtcactaagttccactgcggggttatctacgactactccaccgctgagtacgtaagctaccgtccgagtgacttcggtgcgtatctggatgcgctggaagccgaggttgcacgaggcggtcttattgtgttccacaacggtcacaagtatgacgttcctgcattgaccaaactggcaaagttgcaattgaaccgagagttccaccttcctcgtgagaactgtattgacacc  <  1:351967‑M1/301‑296 (MQ=255)`  
                                                                                                                                                                                                                                                                                                       `gTCACGataatcaataggagaaatcaatatgatcgtttctgacatcgaagctaacgccctcttagagagcgtcactaagttccactgcggggttatctacgactactccaccgctgagtacgtaagctaccgtccaagtgacttcggtgcgtatctggatgcgctggaagccgaggttgcacaaggcagtcttattgtgttccacaacggtcacaagtatgacgttcctgcattgaccaaactggcaaagttgcaattgaaccgagagttccaccttcctcgtgagaactgtattgacacc  >  1:289502‑M1/1‑6 (MQ=255)`  
                                                                                                                                                                                                                                                                                                       `gTCACGataatcaataggagaaatcaatatgatcgtttctgaaatcgaagctaacgccctcttagagagcgtcactaagttccactgcggggttatctacgactactccaccgctgagtacgtaagctaccgtccgagtgacttcggtgcgtatctggatgcgctggaagccgaggttgcacgaggcggtcttattgtgttccacaacggtcacaagtatgacgttcctgcattgaccaaactggcaaagttgcaattgaaacgagagttccaccttcctcgtgagaacctgtcccttatt  >  1:432165‑M1/1‑6 (MQ=255)`  

                                                                                                                                                                                                                                                                                                            `|`                                                                                                                                                                                                                                                                                                         
`GCTACATGGAATACAACAAGGAAACCGGATGGCTTGAACCATCAAGTTACTCAGGGGAAGAAGAGTCACACTCAGAGTCAACAGACTGGTCCAACGACACTGACTTCTGACAGGATTCTTGATGACTTTCCAGACGACTACGAGAAGTTTCGCTGGAGAGTCCCATTCTAATACGACTCACTAAAGGAGACACACCATGTTCAAACTG‑ATTAAGAAGTTAGGCCAACTGCTGGTTCGTATGTACAACGTGGAAGCCAAGCGACTGAACGATGAGGCTCGTAAAGAGGCCACACAGTCACGCGCTCTGGCGATTCGCTCCAACGAACTGGCTGACAGTGCATCCACTAAAGTTACCGAGGCTGCCCGTGTGGCAAACCAAGCTCAACAGCTTTCCAAATTCTTTGAGTAATCAAACAGGAGAAACCATTATGTCTAACGTAGCTGAAACTATCCGTCTATCCGATACAGCTGACCAGTGGAACCGTCGAGTCCACATCAACGTTCGCAACGGTAAGGCGACTATGGTTTACCGCTGGAAGGACTCTAAGTCCTCTAAGAATCACACTCAGCGTATGACGTTGACAGATGAGCAAGC  >  NC_001604.1/13156‑13750`

  

Alignment Legend

Aligned base mismatch/match (shaded by quality score): `ATCG/ATCG` `< 3 ≤` `ATCG/ATCG` `< 11 ≤` `ATCG/ATCG` `< 24 ≤` `ATCG/ATCG` `< 35 ≤` `ATCG/ATCG` `< 38 ≤` `ATCG/ATCG`

Unaligned base: `atcg`    Masked matching base: `atcg`    Alignment gap: `‑`    Deleted base: `‑`

Reads not counted as support for junction

read\_name Not counted due to insufficient overlap past the breakpoint.

read\_name Not counted due to not crossing MOB target site duplication.
